# Fixed Drug Eruption Following Concurrent Ciprofloxacin and Metronidazole Therapy: A Dermoscopy-Assisted Diagnosis

**DOI:** 10.7759/cureus.104084

**Published:** 2026-02-22

**Authors:** Pilar Tuesta Buchelli, David D Terrones Huamán, Karla J Reynel, Julio J Barrios Aedo, Rubi Collahua Cabana

**Affiliations:** 1 Department of Internal Medicine, School of Medicine, Universidad San Martin de Porres, Chiclayo, PER; 2 Department of Internal Medicine, School of Medicine, Universidad Científica del Sur, Lima, PER; 3 Department of Internal Medicine, School of Medicine, Universidad Ricardo Palma, Lima, PER; 4 Department of Internal Medicine, Hospital Guillermo Almenara Irigoyen, Lima, PER

**Keywords:** adverse-drug reaction, ciprofloxacin, delayed hypersensitivity, fixed-drug eruption, fluoroquinolones, metronidazole

## Abstract

Fixed drug eruption (FDE) is a dermatological manifestation characterized by well-circumscribed erythematous-violaceous lesions that recur at identical anatomical sites upon re-exposure to the offending medication. We present a 39-year-old male who developed symmetric erythematous-violaceous macules in bilateral axillary, inguinal, and popliteal regions within 48 hours of initiating concurrent ciprofloxacin and metronidazole therapy for acute infectious diarrhea. Physical examination revealed six well-demarcated, tender, pruritic macules without mucosal involvement or systemic manifestations. Dermoscopic examination demonstrated a homogeneous violaceous background with fine pigment granularity throughout all affected areas. Due to resource limitations in the private clinic setting, skin biopsy and definitive drug causality testing were not performed. Both antibiotics were discontinued upon recognition of the cutaneous reaction. Treatment consisted of systemic corticosteroids (prednisone 20 mg daily for seven days), oral antihistamines (desloratadine 5 mg daily), and topical therapy including fluticasone propionate 0.05% nightly and calamine-based emollients. Symptomatic improvement was achieved with initial emergency treatment. Near-complete resolution of erythema occurred by day nine with residual post-inflammatory hyperpigmentation at previously affected sites. This case highlights the diagnostic challenge when multiple potential culprits are administered simultaneously, and confirmatory testing is unavailable. Dermoscopy proved valuable as a non-invasive diagnostic tool when histopathology was inaccessible. This report emphasizes the importance of clinical vigilance when prescribing commonly used antibiotics and demonstrates the utility of alternative diagnostic approaches in resource-limited settings. Primary care physicians and dermatologists should maintain awareness of FDE presentations and counsel patients to avoid re-exposure to implicated medications.

## Introduction

Fixed drug eruption (FDE) is a distinct type of cutaneous adverse drug reaction characterized by well-circumscribed, erythematous-violaceous plaques that recur at identical anatomical sites upon re-exposure to the offending medication [1-4]. FDE is a delayed, type IV hypersensitivity reaction mainly mediated by CD8+ T lymphocytes [3,5] and represents roughly 16-21% of all cutaneous drug reactions in India [5]. The most frequently implicated drugs involve analgesics, antibiotics, muscle relaxants, and anticonvulsants [2]. Among fluoroquinolones, ciprofloxacin has been reported as a triggering agent in FDE, though it remains relatively uncommon, with adverse skin reactions occurring in only 1-2% of treated patients [2,6]. Likewise, metronidazole-induced FDE has been described in the literature [7], with variable cross-reactivity patterns among nitroimidazole compounds [3].

Clinically, FDE usually presents within 30 minutes to eight hours following drug exposure [3,4], presenting as pruritic, burning erythematous macules that may evolve to bullous lesions [1,2]. The acute phase heals with characteristic residual hyperpigmentation lasting months to years [8]. Common affected areas include extremities, genitals, typically the glans penis, and oral mucosa. [6]. Histopathologically, FDE exhibits vacuolar degeneration of the basal cell layer, necrotic keratinocytes (civatte bodies) in the epidermis, and lichenoid lymphocytic infiltrate with melanin incontinence [1-2,8]

Although ciprofloxacin and metronidazole have each been individually implicated in FDE, cases involving simultaneous use of both antibiotics are rarely documented. Additionally, the diagnostic approach when multiple potential culprits are administered simultaneously, and definitive testing is unavailable, remains undiscussed. We report a case of FDE following combined ciprofloxacin-metronidazole therapy for acute infectious diarrhea, diagnosed by clinical correlation and dermoscopic evaluation in the absence of histopathological confirmation. This case underscores the diagnostic challenge when multiple causative medications are initiated simultaneously and illustrates the practical utility of dermoscopy as a noninvasive diagnostic tool in resource-limited settings.

## Case presentation

A 39-year-old male with no significant medical history presented to the emergency department. On Day 1, the patient initiated ciprofloxacin 500 mg every 12 hours and metronidazole 500 mg every eight hours for acute infectious diarrhea. Approximately 48 hours later (Day 3), erythematous macules first appeared in intertriginous areas, gradually increasing in size and intensity. On Day 5, he presented to the emergency department with a 2-day history of painful, enlarging cutaneous lesions. The patient denied previous similar episodes, recent use of new medications, herbal supplements, or over-the-counter drugs. He also denied fever, respiratory symptoms, oral or genital mucosal involvement, or new systemic manifestations beyond the initial diarrhea. 

**Figure 1:**
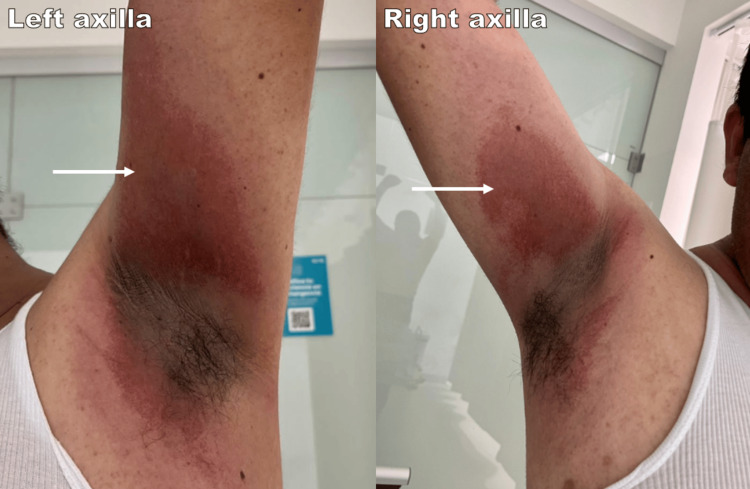
Patient images at initial presentation (Day 5) The images show bilateral well-demarcated erythematous-violaceous patches in the axillary regions.

On admission, vital signs were within normal limits, and the patient was fully alert and oriented. Physical examination revealed six well-demarcated, round to oval erythematous-violaceous macules, symmetrically distributed in bilateral axillary, inguinal folds, and popliteal regions (Figure 1). All lesions were tender to palpation, pruritic, but without evidence of blistering, erosions, or scaling. Oral mucosa, conjunctivae, and genitalia were spared. The remainder of the physical examination was unremarkable.

Laboratory studies showed no systemic abnormalities. Given the temporal association with antibiotic exposure, the characteristic well-circumscribed lesions in fixed locations, along with the absence of systemic involvement, FDE was strongly suspected. The patient received intravenous hydrocortisone 250 mg, chlorphenamine 10 mg, and normal saline 0.9% (100 mL) infused over 20 minutes to achieve rapid symptom control and to mitigate further inflammatory progression while more severe cutaneous drug reactions were excluded. This resulted in significant symptomatic improvement. The patient was discharged with oral cetirizine 10 mg daily, and dermatology follow-up was scheduled within the same week. At dermatology consultation, dermoscopy revealed a homogeneous violaceous background with fine pigment granularity, findings consistent with FDE. Based on morphology, distribution, temporal association, and dermoscopic features, the clinical presentation was most consistent with FDE, and both ciprofloxacin and metronidazole were considered suspected triggers.

**Figure 2:**
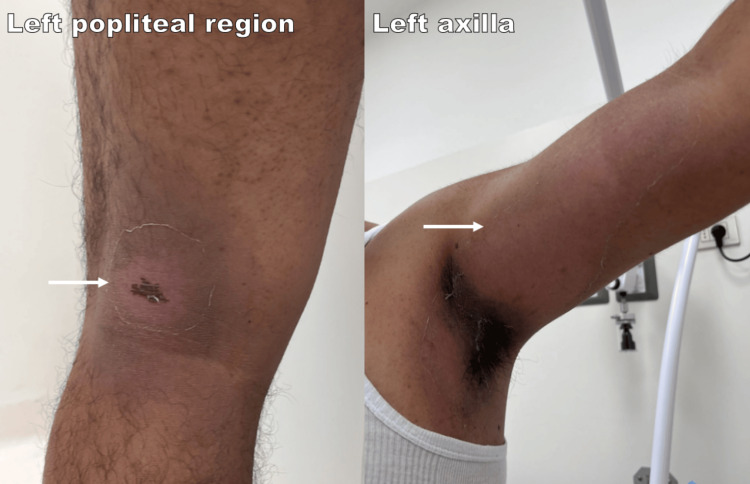
Post-inflammatory hyperpigmentation in the patient The images show post-inflammatory hyperpigmentation in the popliteal region and axilla at Day 14 (nine days after initial presentation).

Treatment was initiated with prednisone 20 mg daily for seven days, and desloratadine 5 mg daily for seven days. The sequential use of antihistamines, chlorphenamine in the acute setting, followed by cetirizine at discharge and desloratadine during outpatient follow-up, reflected a stepwise de-escalation approach to maintain symptom control while improving tolerability for ambulatory care. In addition, a topical regimen was initiated, including calamine with zinc, hyaluronic acid, and panthenol every eight hours, plus fluticasone propionate 0.05% nightly for one month. Partial resolution occurred by Day 14 (nine days after initial presentation), with expected persistent post-inflammatory hyperpigmentation (Figure 2). The patient avoided re-exposure to both agents and remained clinically stable.

## Discussion

This case describes a fixed drug eruption (FDE) temporally linked to co-administration of ciprofloxacin and metronidazole therapy for acute infectious diarrhea. The combination of ciprofloxacin and metronidazole was prescribed empirically when mixed bacterial and protozoal etiology was suspected. Although current guidelines reserve empiric fluoroquinolone therapy for adults with features of invasive bacterial infection [9], metronidazole is recognized as first-line antiprotozoal coverage against *Entamoeba histolytica* [10]. The specific clinical features that guided this therapeutic decision were not available for documentation, which represents a limitation of this case report. The cutaneous presentation of well-demarcated, symmetric erythematous-violaceous macules appearing 48 hours after drug initiation is compatible with FDE, a delayed-type hypersensitivity reaction mediated by resident epidermal CD8+ T cells [3,5]. 

Among fluoroquinolones, ciprofloxacin has been documented to trigger FDE, occurring in 1-2% of treated patients [2,6]. Nair et al. reported three cases of ciprofloxacin-induced bullous FDE with quick progression and blister formation [2]. Differing from those observations, our patient showed no evidence of bullous lesions despite similar onset timing, indicating variation in severity even with the same offending agent. Iliyas et al. described a widespread non-bullous FDE to ciprofloxacin with trunk involvement [6], whereas our case showed predominant flexural distribution sparing the trunk, emphasizing the heterogeneous presentation patterns of ciprofloxacin-induced FDE.

Metronidazole, though less frequently documented, has also been implicated in FDE. Kumar et al. described metronidazole-induced FDE with typical reappearance at the same anatomical site [7]. Interestingly, Pal et al. identified variable cross-reactivity patterns within the nitroimidazole class, reporting cases in which patients reacted to tinidazole and ornidazole but were unaffected by metronidazole, attributed to chemical variation in molecular side chains [3]. This variability underlines the difficulty of predicting cross-reactivity within this drug class and reinforces our management strategy to suggest long-term avoidance of both implicated agents. Notably, Kameswari et al. showed cross-reactivity among fluoroquinolones [5]. This has critical clinical implications since patients sensitized to ciprofloxacin may experience similar reactions when exposed to other fluoroquinolones, necessitating avoidance of the entire drug class, a key counseling point in our patient's management. 

The concurrent administration of two potential culprits presents a significant diagnostic challenge in definitively identifying the causative agent. Oral provocation testing, considered the gold standard for diagnosis [1], and patch testing [1,2], were not performed in this case due to resource limitations in the private clinic setting and the potential risk of inducing a more severe reaction. Therefore, both medications were empirically avoided as a precautionary measure, a pragmatic approach when multiple drugs are suspected, and definitive testing is unavailable. To formally assess causality, the Naranjo Adverse Drug Reaction Probability Scale was applied [11]. The patient scored 1 point, corresponding to a "possible" adverse drug reaction, reflecting the temporal association with drug initiation, offset by the concurrent administration of two potential culprits, the absence of isolated drug withdrawal without simultaneous pharmacological treatment, and the lack of rechallenge confirmation.

Dermoscopic examination revealed a homogeneous violaceous to brown background with fine pigment granularity, findings consistent with melanin deposition at different levels of the epidermis and dermis, characteristic of FDE [12]. These dermoscopic features helped differentiate FDE from other cutaneous drug reactions such as erythema multiforme, bullous disorders, and toxic epidermal necrolysis [6,12]. Symmetric Drug-Related Intertriginous and Flexural Exanthema (SDRIFE) was also considered, given the symmetric flexural distribution following systemic antibiotic exposure [13]. However, SDRIFE typically presents as a diffuse erythema without well-demarcated borders and does not produce residual post-inflammatory hyperpigmentation, in contrast to FDE, which characteristically leaves pigmented lesions [13]. Furthermore, our patient's dermoscopic findings of violaceous pigmentation with fine granularity reflect melanin deposition consistent with FDE rather than SDRIFE. The characteristic symmetric flexural distribution [12], well-demarcated erythematous-violaceous morphology, and absence of widespread mucosal involvement or systemic toxicity [2] favored FDE over these alternative diagnoses. The dermoscopic findings proved particularly valuable in supporting diagnosis when histopathological confirmation was unavailable, demonstrating the utility of this non-invasive technique in resource-limited settings.

Unlike the generalized presentation described by Iliyas et al. [6], our patient demonstrated a more localized flexural distribution. The absence of mucosal involvement in our case contrasts with reports where oral or genital mucosa were affected [1,2], further illustrating the spectrum of FDE presentations. The rapid response to corticosteroids and complete avoidance of bullous progression distinguish our case from the more severe bullous variants reported by Nair PA [2] and Jain and Jain [8].

The absence of histopathological confirmation limits absolute diagnostic certainty, though clinical and dermoscopic findings were highly characteristic. Without rechallenge or patch testing, definitive causality attribution to a single agent remains impossible. Despite these limitations, this case contributes valuable documentation of FDE following concurrent ciprofloxacin-metronidazole administration, demonstrates the practical utility of dermoscopy when histopathology is unavailable, and provides guidance for managing FDE when multiple potential culprits are involved and definitive testing is not feasible.

## Conclusions

This case underscores the importance of being aware of both ciprofloxacin and metronidazole as potential FDE triggers, including those with no history of hypersensitivity. Co-administration of both antibiotics makes it difficult to identify the culprit; while ciprofloxacin is more commonly associated and demonstrates cross-reactivity within the fluoroquinolone class, metronidazole is also a viable candidate based on this case presentation. Differing from prior reports of bullous or generalized involvement, our patient exhibited localized non-bullous lesions in flexural areas, illustrating the heterogeneity in clinical features. Dermoscopy showed great utility as a non-invasive diagnostic tool in low-resource environments where histopathology is unavailable. Prompt drug withdrawal led to rapid improvement, emphasizing the critical importance of early recognition and management.
